# Supplementary material for: Local inversion-symmetry breaking in a bismuthate high-Tc superconductor
Source: Nat Commun. 2023 Feb 15;14:845. doi: 10.1038/s41467-023-36348-9 (PMC9931700; doi:10.1038/s41467-023-36348-9)
Supplement: Supplementary file 1 — Supplementary Information [file 41467_2023_36348_MOESM1_ESM.pdf]

Supplementary Information for

Local inversion-symmetry breaking in a bismuthate high-T_c superconductor

S. Griffitt[1*#], M. Spaić[2*], J. Joe[1], Z. Anderson[1], D. Zhai[1], M. J. Krogstad[3], R. Osborn[3], D. Pelc[1,2†], and M. Greven[1†]

[1]School of Physics and Astronomy, University of Minnesota, Minneapolis, MN 55455, USA

[2]Department of Physics, Faculty of Science, University of Zagreb, Bijenička 32, HR-10000 Zagreb, Croatia

[3]Materials Science Division, Argonne National Laboratory, Lemont, IL 60439, USA

*authors contributed equally

#present address: Cornell University, Ithaca, NY 14850, USA

†correspondence to: dpelc@phy.hr, greven@umn.edu

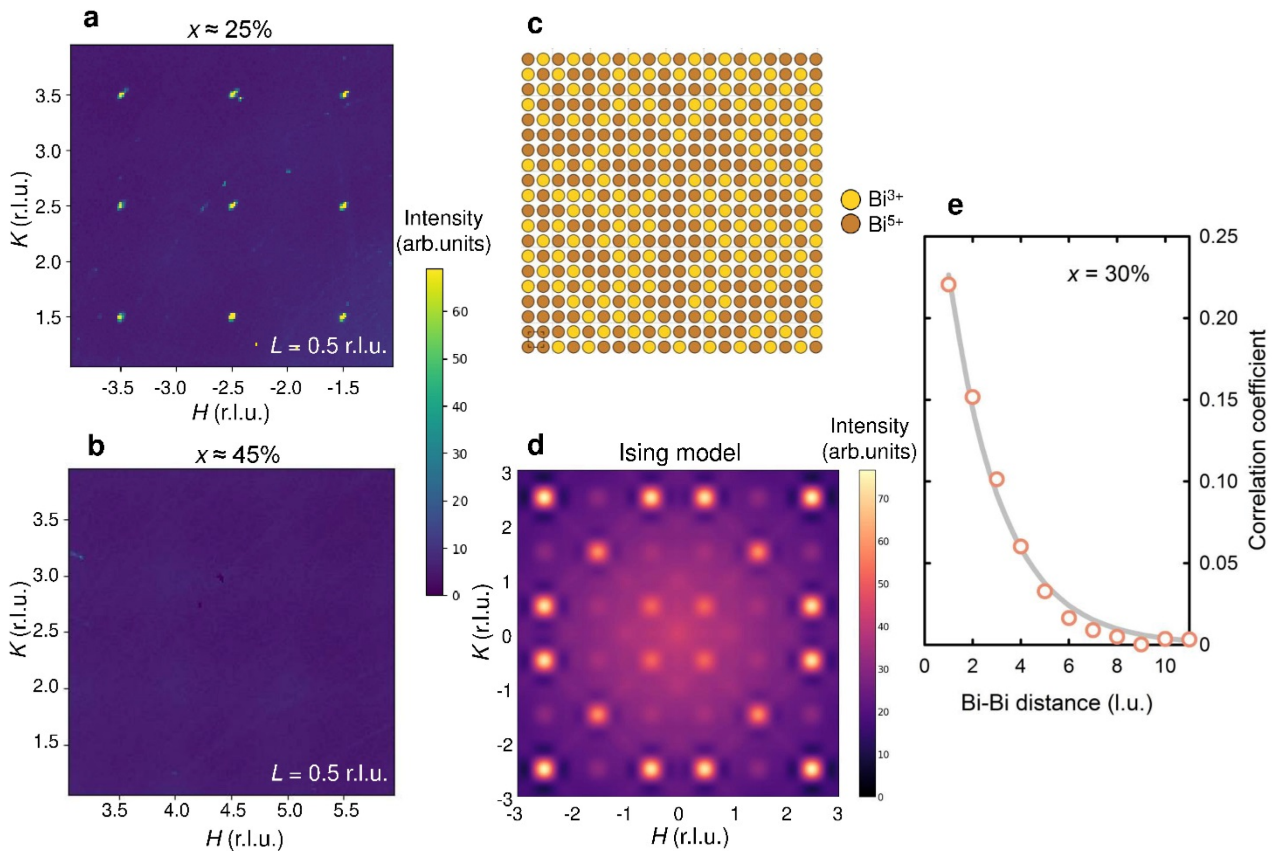

Supplementary Fig. S1 | Short-range charge-density-wave features. a,b, X-ray scattering data for BKBO with doping levels $x \approx 25\%$ and 45%, respectively, in the $L = ½$ plane; r.l.u. refers to reciprocal lattice units. If short-range CDW correlations were present, broad diffuse peaks at half-integer positions would be observed. Such features are absent in both insulating (**a**) and metallic (**b**) samples. Instead, sharp superstructure peaks are seen in **a** due to a known long-range tilt distortion that is unrelated to the CDW and becomes very weak in the metallic/superconducting doping range [20]. The superstructure peaks appear at (½ ½ ½) and all equivalent positions due to the presence of tetragonal domains in the tilted phase; the tilt distortion in a single domain intrinsically does not double the lattice parameter, in contrast to the CDW. **c,** Typical equilibrium distribution of effective Ba^{3+} and Ba^{5+} ions in a simple Bi valence Ising model with frustration (one Monte Carlo relaxed configuration – see Methods for details). The K concentration is 30%. **d,** Diffuse scattering for the same plane as in **a** and **b**, calculated from Monte Carlo simulations of the Ising model with included oxygen breathing distortions, with effective K concentration of 35%. The model contains short-range CDW correlations which clearly lead to diffuse features, in stark contrast to the measured diffuse scattering. **e**, Short-range CDW correlation length in the Ising model with effective K concentration of 30%, calculated from a simple exponential fit to the correlation coefficient c_{ij}, Eq. (4) (Methods). The correlation length is 2.2 ± 0.1 lattice units (error is SD from fit).

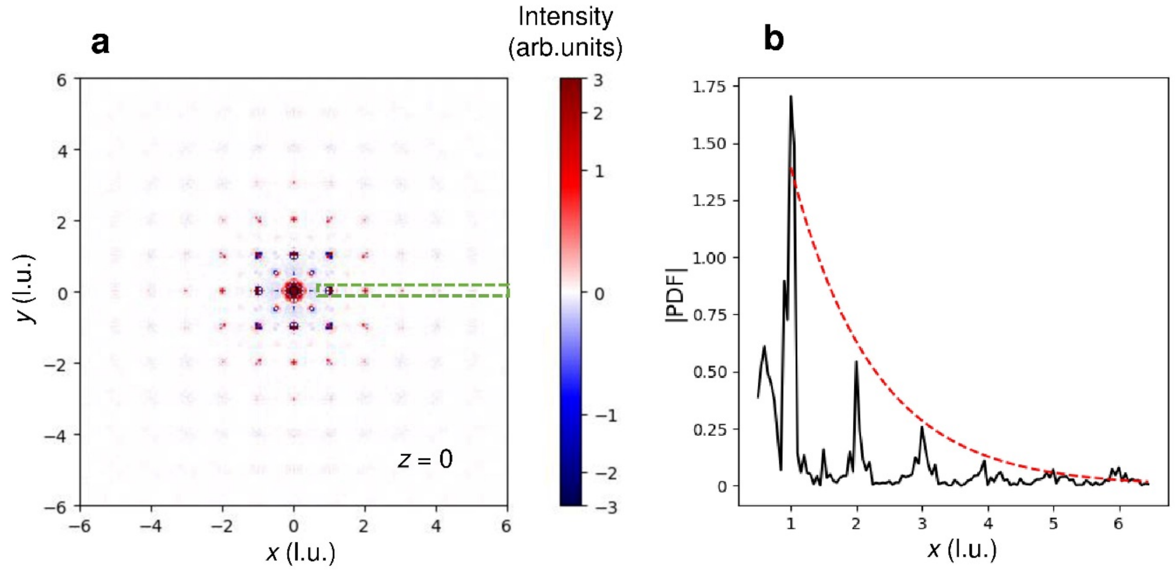

Supplementary Fig. S2 | Structural correlation length. a, Expanded view of the 3D-ΔPDF in Fig. 2c, showing the decay of correlations with increasing pair distance; l.u. refers to lattice units. **b,** The characteristic length for short-range correlations is obtained by fitting a simple exponential decay to the absolute value of the 3D-ΔPDF (sample with $x \approx 0.45$, 30 K). The representative cut shown here is $y/a = 0$, $z/a = 0$ (dashed box in **a**), and the decay constant from the fit is 1.1 ± 0.1 l.u. (error is SD from fit), which implies that the signal decays significantly within 2-3 unit cells (about 1 nm).

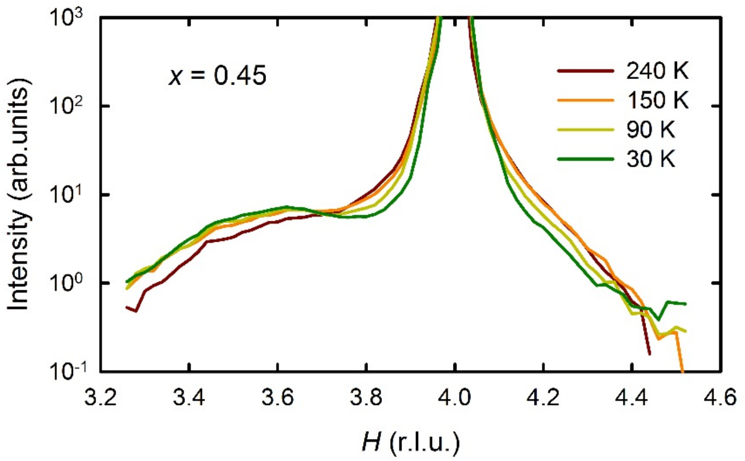

Supplementary Fig. S3 | Temperature evolution of symmetric and asymmetric diffuse scattering. One-dimensional line cuts ($L = K = 0$) through the data at four temperatures for the $x \approx 45\%$ sample; r.l.u. refers to reciprocal lattice units. The intensity of the asymmetric component that is predominantly visible on the low-wavevector side of the (4 0 0) Bragg peak decreases somewhat with temperature, while the intensity of a symmetric, likely phonon-related component increases.

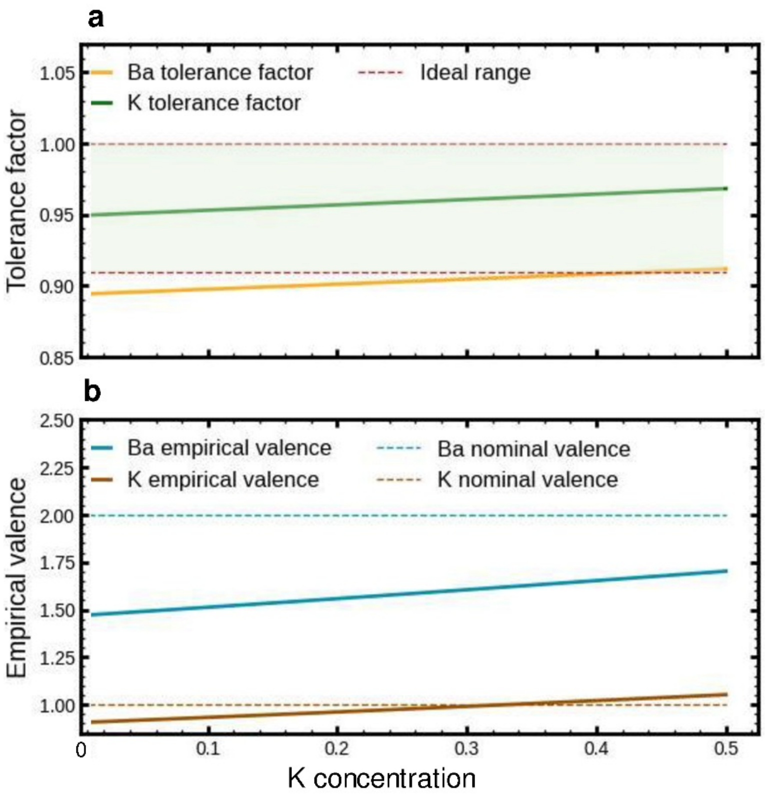

Supplementary Fig. S4 | Structural and chemical parameters of Ba and K in the local-structure model. **a,** Perovskite tolerance factor t (see Methods), showing that the K ion size matches the ideal perovskite structure (t = 1) better than Ba, with further improvement upon doping. The shaded 'ideal range' is the approximate range where the cubic perovskite structure can be stabilized. The Ba size mismatch predominantly leads to rigid tilt distortions, which decrease with increasing K concentration. **b,** Empirical valence of Ba and K (see Methods), demonstrating that K is very close to the nominal valence of +1, whereas Ba is quite strongly underbonded. This leads to an effective attraction between Ba and O, which results in inversion-breaking displacements of the oxygen atoms in local environments with an asymmetric arrangement of Ba and K.

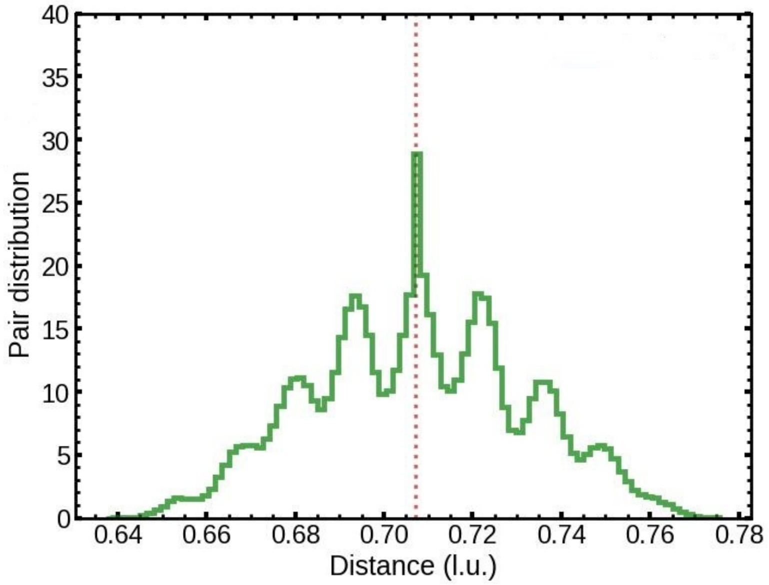

Supplementary Fig. S5 | Calculated oxygen-oxygen correlations. Distances between nearest-neighbour pairs of oxygen atoms obtained from Monte Carlo simulations (see Methods for details), similar to Fig. 3**b,c**. The vertical dashed line indicates the distance in the average structure. The individual peaks roughly correspond to inversion-breaking distortions in different local environments, *e.g.*, with different local Ba/K ratios and spatial configurations.

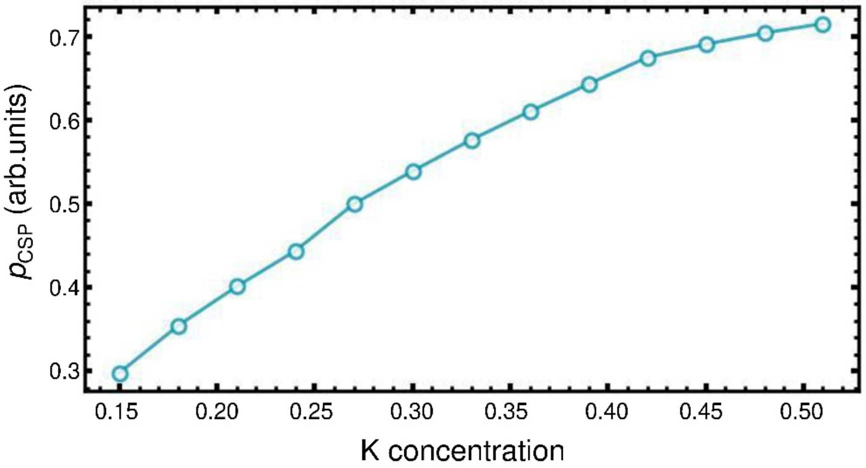

Supplementary Fig. S6 | Calculated K concentration dependence of the inversion-breaking distortion. The centrosymmetry parameter p_{CSP} (see Methods), used as a direct measure of the inversion-breaking displacements, shows a substantial increase with K concentration, in qualitative agreement with the experimental results. The modeling does not take the insulator-metal transition into account, *i.e.*, we use screened electrostatic interactions appropriate for the metallic phase throughout.
